# Comparing the Effect of Osseodensification Versus Conventional Drilling Technique on Implant Stability and Bone Width in the Alveolar Ridge Split Procedure: A Retrospective Study

**DOI:** 10.3390/jcm14207431

**Published:** 2025-10-21

**Authors:** Yunus Emre Guner, Varol Canakci

**Affiliations:** Department of Periodontology, Faculty of Dentistry, Ordu University, Ordu 52200, Turkey; varolcanakci@gmail.com

**Keywords:** osseodensification, alveolar ridge split, dental implants, implant stability, cone-beam computed tomography (CBCT), resonance frequency analysis (RFA), Implant Stability Quotient (ISQ)

## Abstract

**Background/Objectives:** Horizontal alveolar ridge deficiency is a common clinical challenge in dental implant placement. The osseodensification (OD) technique has been proposed as a minimally invasive alternative to conventional osteotomy. This study aimed to compare the outcomes of OD and conventionally performed ridge-split procedures in terms of implant stability and horizontal bone gain. **Methods:** In this retrospective study, 65 patients (a total of 268 implants) who underwent simultaneous implant placement with ridge-split procedures were evaluated. Cases were divided into two groups: OD burs (n = 133 implants) and the conventional Esset kit (n = 135 implants). Ridge width was measured at coronal and apical levels using cone-beam computed tomography (CBCT) preoperatively and four months postoperatively. Implant stability was assessed at the time of placement (primary stability) and at four months (secondary stability) using resonance frequency analysis (RFA). **Results:** Both techniques achieved comparable horizontal bone gain (1.1–1.6 mm; *p* > 0.05). In the maxilla, the OD group demonstrated a tendency toward higher primary and secondary stability values (*p* < 0.01). A similar trend was observed for secondary stability in the mandibular posterior region (*p* < 0.01). The mean Implant Stability Quotient (ISQ) values in the OD group generally exceeded the threshold of 65, considered sufficient for prosthetic loading. **Conclusions:** The findings suggest that the OD technique may have a favorable effect on implant stability, particularly in regions with low-to-moderate bone density, while providing comparable horizontal bone gain to the conventional method. These results indicate that OD could serve as a potentially useful alternative in the management of horizontal ridge deficiencies; however, its long-term efficacy should be further evaluated in larger, prospective clinical studies.

## 1. Introduction

Dental implants are currently recognized as a reliable and predictable treatment option for the rehabilitation of partially or completely edentulous patients [1,2]. This reliability derives from the successful osseointegration of biocompatible materials such as titanium with bone, as well as the superior biomechanical properties of implants [3]. A successful implant-supported restoration depends not only on the prosthetic design but also on the harmonious integration of the surrounding hard and soft tissues [4,5]. Therefore, to achieve optimal esthetic and functional outcomes, implants must be positioned three-dimensionally in a correct location, considering both bone and soft tissue volume [6,7].

The concept of osseointegration was introduced to the literature by Brånemark and defined as a direct and rigid connection between the implant surface and living bone without any intervening soft tissue layer [8]. At the onset of this biomechanical bond, primary stability plays a critical role, whereas secondary stability becomes essential during the healing phase [9]. Primary stability refers to the mechanical engagement of the implant with the bone at the time of placement, while secondary stability reflects the biological healing process, involving bone resorption and new bone formation [10,11,12]. Conventional osteotomy techniques prepare the implant socket by removing bone tissue, which may result in reduced bone volume and compromised primary stability [13,14].

To overcome these limitations, the osseodensification (OD) technique was introduced in 2015 by Huwais and Meyer [13]. This approach, performed using “Densah” burs, increases the density of the osteotomy walls by compacting rather than removing bone [15]. While these burs function as traditional drills in a clockwise direction, when operated counterclockwise they redirect autogenous bone particles toward the osteotomy walls and apical region, thereby enhancing both lateral and apical bone density. Preclinical studies have demonstrated that implant insertion torque, bone-to-implant contact, and early mechanical stability are significantly higher in sockets prepared with OD compared to conventional techniques [13,16].

One of the major challenges in implant planning is horizontal alveolar ridge deficiency [17]. The alveolar ridge split technique, initially described by Tatum and later refined by Summers and Khoury, is based on the controlled separation of the buccal plate to allow expansion and graft placement [18,19,20]. However, especially in the mandible, the presence of dense cortical bone increases the risk of fracture and complicates the achievement of primary implant stability [21,22].

The literature regarding the effectiveness of the osseodensification technique remains limited, with most studies focusing on in vitro or animal models. Clinical research is generally restricted to short-term case reports with small sample sizes. Furthermore, many investigations have evaluated the role of OD in sinus augmentation procedures, whereas clinical evidence on ridge expansion is still insufficient. We hypothesized that dental implants placed via osteodensification may have a more positive effect on implant stability and bone width than the conventional drilling technique and may affect long-term success. Thus, the aim of this study is to compare the effects on alveolar ridge width and implant stability of osseodensification and conventional drilling techniques in the alveolar ridge split procedures of patients with horizontal ridge deficiency.

## 2. Materials and Methods

### 2.1. Study Design and Patient Selection

The retrospective study was conducted with the approval of the Ordu University Clinical Research Ethics Committee (Approval No: 2023/268, dated 13 October 2023) and in accordance with the latest revisions of the Declaration of Helsinki. Written informed consent was obtained from all participants. All clinical and radiographic data were anonymized prior to analysis to ensure patient confidentiality.

A total of 65 patients (34 males, 31 females; age range 31–69 years, mean age 51.8 years) who underwent simultaneous dental implant placement with alveolar ridge split procedures at the Department of Periodontology, Faculty of Dentistry, Ordu University between 30 July 2021, and 1 January 2023, and had complete clinical and radiological records were included. A total of 268 implants were retrospectively evaluated. To assess the effects of osseodensification and conventional drilling techniques across different bone qualities, cases were categorized according to anatomical region rather than individual CBCT Hounsfield unit (HU) values. The maxillary anterior, maxillary posterior, mandibular anterior, and mandibular posterior regions were analyzed separately, reflecting the typical bone density distribution described by Misch. Accordingly, the mandibular anterior region generally corresponds to D1–D2 bone, while the maxillary posterior region represents D3–D4 bone, with other regions indicating intermediate densities. This anatomical classification was used as a clinically meaningful proxy for baseline bone quality, particularly in cases lacking standardized HU data. Of the 268 implants, 82 were placed in the maxillary anterior, 82 in the maxillary posterior, 82 in the mandibular posterior, and 22 in the mandibular anterior regions. Patients were retrospectively divided into groups based on the surgical technique:

- Test group (n = 133 implants): Ridge split and implant placement using osseodensification (OD) burs (Densah™, Versah LLC, Jackson, MI, USA).
- Control group (n = 135 implants): Ridge split and implant placement using the conventional Esset kit (Osstem Implant Co., Seoul, Republic of Korea) and standard drills.

Inclusion criteria: Patients ≥ 18 years of age; ASA I–II classification; good oral hygiene; complete clinical and radiological records.

Exclusion criteria: Patients < 18 years of age; ASA III–VI classification; poor oral hygiene; incomplete records; systemic contraindications to surgery; alcohol or nicotine dependence.

### 2.2. Surgical Protocol

All surgical procedures were performed under aseptic conditions. Extraoral antisepsis was achieved with 10% povidone–iodine (Konix, Istanbul, Turkey), and local anesthesia was administered using articaine HCl with epinephrine (Ultracain DS, Sintetica, Turkey). A mucoperiosteal flap was elevated in all cases, and osteotomies were prepared according to group allocation.

**Figure 1:**
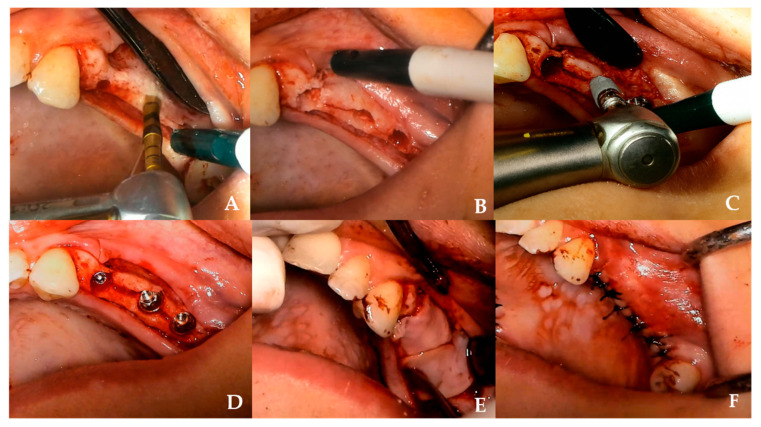
Clinical sequence of ridge-split with OD technique. (**A**) Use of a Densah^®^ bur in osseodensification mode under irrigation to initiate the osteotomy. (**B**) Plastic deformation of the alveolar ridge observed after osteotomy preparation with Densah^®^ burs. (**C**) Placement of an appropriately sized implant into the expanded osteotomy site. (**D**) Immediate implant installation following ridge expansion. (**E**) Placement of a CGF membrane over the operation site prior to flap closure. (**F**) Primary closure achieved with tension-free interrupted sutures.

**Figure 2:**
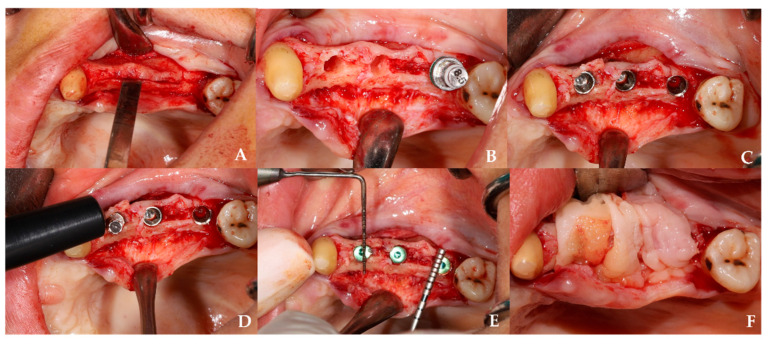
Clinical sequence of ridge-split with conventional technique. (**A**) Ridge-split procedure performed using chisels and mallet to create cortical separation. (**B**) Post-expansion view of the alveolar crest following the use of expansion drills. (**C**) Placement of implants into the expanded ridge after achieving sufficient cortical separation. (**D**) Measurement of the primary stability of the placed implants using an Osstell device. (**E**) Postoperative measurement of ridge width using a periodontal probe. (**F**) Placement of a CGF membrane over the operation site prior to flap closure.

- Test group (OD): A pilot osteotomy was initiated with a Densah bur at 800 rpm in clockwise mode. Subsequently, osseodensification was performed in counterclockwise mode at 1200 rpm using burs of increasing diameters (2.3–3.3 mm) [Figure 1].
- Control group: Osteotomies were prepared with twist and expansion burs from the Osstem Esset ridge split kit (1.6–4.1 mm) at 800–1200 rpm, using conventional cutting and pumping motions (Figure 2).

In both groups, implants (Osstem Implant Co., Seoul, Republic of Korea) were placed using a surgical motor system (NSK Surgic Pro, Nakanishi Inc., Tokyo, Japan). Peri-implant gaps covered with concentrated growth factor (CGF) membranes. Flaps were closed with 3-0 or 4-0 silk sutures (Dogsan, Trabzon, Turkey).

Postoperative care included systemic antibiotic therapy, analgesics, and standardized oral hygiene instructions. All surgeries were performed by the same experienced periodontist to minimize operator-related variability.

### 2.3. Clinical Measurements

**Figure 3:**
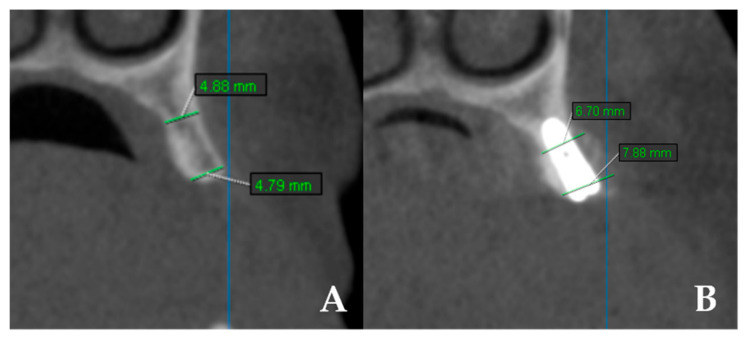
CBCT images showing linear measurements of ridge width before and after surgery. (**A**) Preoperative measurements of horizontal ridge width. (**B**) Postoperative measurements of horizontal ridge width.

- Bone width: CBCT scans (KaVo OP 3D Vision, Imaging Sciences International LLC, Hatfield, PA, USA) were obtained preoperatively and at 4 months. Measurements were performed at coronal and apical levels using OnDemand 3D App v1.0 (Cybermed Inc., Daejeon, Republic of Korea). The difference between baseline and 4 months was recorded as “bone gain” (Figure 3). All measurements were performed twice by a single examiner, with intra-observer reliability exceeding 90%.

- Implant stability: Primary stability (at placement) and secondary stability (at 4 months, during healing abutment connection) were assessed using resonance frequency analysis (RFA) with the Osstell device (Osstell AB, Göteborg, Sweden) and implant-specific SmartPeg transducers. ISQ values were recorded in both mesiodistal and buccolingual directions, each measured twice and averaged.

### 2.4. Statistical Analysis

All data were analyzed using NCSS 2007 software (NCSS, LLC, Kaysville, UT, USA). The normality of the data distribution was assessed using the Shapiro–Wilk test. Descriptive statistics, including mean, standard deviation, median, minimum, and maximum values, were reported. Independent samples *t*-tests or Mann–Whitney U tests were used to compare independent groups, while paired samples *t*-tests or Wilcoxon signed-rank tests were applied for within-group comparisons. For multiple group comparisons, one-way ANOVA or Kruskal–Wallis tests were employed, followed by Bonferroni correction for post hoc analysis. Statistical significance was set at *p* < 0.05.

An a priori power analysis was conducted using G*Power 3.1 software (Heinrich Heine University, Düsseldorf, Germany) to estimate the minimum required sample size for detecting differences between the osseodensification (OD) and conventional groups. The analysis was based on the implant stability quotient (ISQ) values from the maxillary anterior region, which showed the largest intergroup variation (OD: 81.98 ± 4.0; control: 77.37 ± 5.5; *p* = 0.001). The corresponding effect size (Cohen’s *d*) was calculated as 0.90, representing a large effect. Using a two-tailed independent samples *t*-test with an alpha level of 0.05 and a desired power of 0.90 (1–β), the required sample size was determined as 36 implants in total (18 per group). Given the actual sample size of 133 implants in the OD group and 135 implants in the control group, the achieved post hoc power exceeded 0.99, confirming that the study was sufficiently powered to detect clinically meaningful differences in implant stability.

## 3. Results

### 3.1. Demographic and Clinical Characteristics

A total of 65 patients (34 males, 31 females; mean age 51.8 ± 9.2 years; range 31–69) and 268 implants were analyzed.

All patients were classified as ASA I–II, and complete surgical, radiological, and clinical records were available.

### 3.2. Implant Stability (ISQ)

In this study, implant stability measurements were evaluated separately within distinct anatomical regions, namely the maxillary anterior, maxillary posterior, mandibular anterior, and mandibular posterior areas. Primary (T0) and secondary (T1) stability values of each implant were recorded, and comparisons were made between the osseodensification and control groups. Statistical analyses were performed to determine significant differences between groups and stability time points.

In the maxillary anterior and posterior regions, both primary (T0) and secondary (T1) stability values were significantly higher in the OD group compared with the control group (*p* = 0.001; *p* < 0.01) [Table 1].

In the mandibular anterior region, no statistically significant difference was observed between the groups at either T0 or T1 (*p* > 0.05) [Table 1].

In the mandibular posterior region, primary stability (T0) did not differ significantly between groups (*p* > 0.05), whereas secondary stability (T1) was significantly higher in the OD group (*p* = 0.001; *p* < 0.01) [Table 1].

Follow-up RFA measurements revealed no significant differences between T0 and T1 values in the maxillary anterior, maxillary posterior, and mandibular anterior regions for either group (*p* > 0.05). However, in the mandibular posterior region, a significant increase in T1 values was observed only in the OD group (*p* = 0.021), whereas no significant change was detected in the control group (*p* > 0.05) [Table 2].

### 3.3. Bone Width and Bone Gain

In this study, horizontal ridge width was measured at both the coronal and apical levels. Tomographic measurements were recorded in millimeters at three time points: preoperatively (preop), postoperatively (postop), and as bone gain (difference between the two). To compare the osseodensification and control groups, bone gain values (mm) were calculated by subtracting the preoperative ridge width from the postoperative measurements. Statistical analyses were then performed to assess significant differences in bone gain between the groups and between the coronal and apical regions.

No statistically significant differences were observed between the groups regarding preoperative ridge width at coronal and apical levels (*p* > 0.05). This finding indicates that both groups initially presented with comparable ridge widths, suggesting that the comparisons were conducted under equivalent baseline conditions. Regarding bone gain, the mean increase in the coronal region was 2.81 mm in the OD group and 2.99 mm in the control group. In the apical region, the mean bone gain was 0.73 mm in the OD group and 1.37 mm in the control group. Although the control group exhibited slightly greater bone gain than the OD group at both the coronal and apical levels, the differences were not statistically significant (*p* > 0.05) [Table 3].

**Figure 4:**
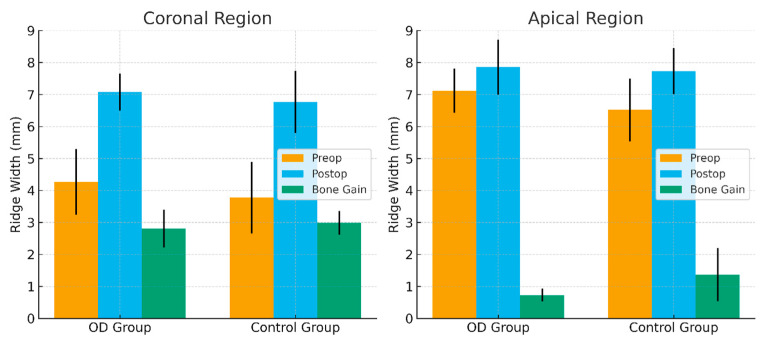
Comparison of Preoperative, Postoperative Ridge Width and Bone Gain Between OD and Control Groups.

However, intragroup comparisons revealed that both the OD and control groups demonstrated significantly greater bone gain at the coronal level compared with the apical level (*p* = 0.001; *p* < 0.01) [Table 3] (Figure 4).

## 4. Discussion

Dental implants are widely recognized as an effective treatment modality for replacing missing teeth without compromising adjacent structures, and they are generally associated with high long-term success rates. However, the rapid and sometimes insufficiently planned increase in implant procedures has been accompanied by a rise in complication rates, reported to range between 5% and 11% in the literature [23]. One of the principal factors contributing to these complications is thought to be insufficient osseointegration [24]. Primary stability is considered a prerequisite for achieving secondary stability and is regarded as a key determinant of implant success [25].

Bassetti et al. [21] have suggested that a minimum alveolar ridge width of approximately 6 mm may be required for the placement of a standard-sized implant. In the present study, cone-beam computed tomography (CBCT) measurements indicated that residual ridge widths ranged from 2.7 to 5.8 mm, suggesting the need for augmentation procedures. Among the available horizontal augmentation techniques, ridge-split and autogenous block grafting are commonly employed [26]. However, as block grafting often requires an additional donor site, it may increase morbidity and patient discomfort [27,28]. Therefore, the ridge-split approach was selected for the present study. Altıparmak et al. [29] have also reported cases where this technique yielded higher implant survival rates compared with block grafting.

In the present investigation, ridge-split procedures performed with conventional and osseodensification (OD) drilling techniques were retrospectively compared. Implant stability was assessed using resonance frequency analysis (RFA), whereas horizontal bone gain was evaluated using CBCT. RFA is widely accepted as a reliable and objective method for quantifying both primary and secondary stability [30,31]. The findings indicated that mean ISQ values tended to be higher in the mandibular regions than in the maxilla, which may be attributed to the relatively greater bone density typically observed in the mandible.

Various approaches—including osteotomes, condensation drills, undersized drilling, and bicortical fixation—have been proposed to enhance stability in low-density bone [32,33,34]. Nevertheless, some of these methods may prolong operative time or increase surgical trauma. Introduced by Huwais in 2017, the osseodensification technique differs from conventional drilling in that it compacts rather than removes bone, which may in turn enhance the density of osteotomy walls. This process has been proposed to reduce peri-implant defects and potentially improve bone–implant contact [13,35].

Most available studies on OD remain preclinical or limited to short-term case series. However, the current findings, together with those reported by other authors [36,37], suggest that both primary and secondary stability tend to be higher with OD compared to conventional drilling. In particular, a more pronounced improvement was observed in the maxillary regions, while no significant difference was noted in the anterior mandible. This variation could largely be explained by regional differences in bone structure and biological dynamics. The anterior mandible, typically characterized by dense D1-type cortical bone, is mechanically rigid but exhibits limited microvascularization and osteogenic activity. As a result, the biomechanical advantages achieved through OD-induced compaction might not necessarily translate into equivalent biological outcomes in this region [15]. The increase in secondary stability observed in the mandibular posterior region may be explained by regional variations in bone density and cortical structure. The denser D2-type bone commonly found in this area may enhance the compaction and condensation effects achieved by OD, thereby improving implant–bone contact during the healing process. However, considering the complex interplay between biomechanical and biological factors, this finding should be interpreted with caution and confirmed through further site-specific studies.

Jarikian et al. (2021) compared OD and screw-type expander techniques in narrow ridges and found that mean ridge expansion was significantly higher in the OD group [38]. The authors attributed this to the bone-compaction effect of the technique. In the present study, the control group exhibited a tendency toward greater ridge width gain than the OD group, although the difference was not statistically significant. These findings, when considered collectively, may indicate that OD has the potential to promote ridge expansion under certain clinical conditions; however, this effect does not appear to be consistently reproducible.

CBCT measurements demonstrated comparable horizontal bone gains between the two techniques (1.1–1.6 mm). These results are in agreement with those reported by Tofan et al. (2024), who observed similar bone augmentation but noted a longer surgical time in the conventional drilling group [39]. Although surgical time was not quantitatively assessed in the present study, the operating clinician subjectively perceived a shorter duration in the OD group, consistent with previous reports [39]. Taken together, these findings suggest that OD and conventional techniques may yield comparable volumetric outcomes, while OD might offer advantages in terms of reduced invasiveness and shorter surgical duration. Clinically, OD may provide benefits beyond implant stability, including improved surgical efficiency, decreased trauma, and enhanced patient comfort—particularly in elderly or medically compromised patients, or in those less tolerant of prolonged surgical procedures.

Both techniques exhibited a tendency toward greater expansion at the coronal compared with the apical level. This observation aligns with the findings of Koutouzis et al. (2019), who suggested that the difference may relate to drill design and local anatomy [40]. Moreover, the ability of OD to minimize the need for traumatic osteotomes could reduce complication risk and support more favorable healing dynamics [41,42].

Recent randomized clinical trials have provided more detailed insights into the effects of OD on implant stability. Bergamo et al. (2021) evaluated both insertion torque and ISQ values to assess primary and secondary stability in their study, which included 150 dental implants [12]. Similarly to the present study, the jaws were divided into four anatomical regions—maxillary anterior, maxillary posterior, mandibular anterior, and mandibular posterior—and the data were analyzed within these subgroups. The authors reported that the osseodensification (OD) technique yielded higher insertion torque and stability values compared with conventional drilling. A transient decrease in ISQ values was observed in both groups during the third week; however, this reduction was less pronounced in the OD group, with values remaining above a minimum of 68. Consistently, in the present study, the minimum primary stability values were 62 in the control group and 68 in the OD group. For secondary stability, the minimum ISQ values were 61 and 71, respectively. Notably, in the OD group, none of the implants demonstrated ISQ values below the generally accepted threshold for prosthetic loading (ISQ ≥ 65). This finding may suggest that the OD technique has the potential to induce a distinct early remodeling response at the bone–implant interface, thereby contributing to improved short-term stability.

Similarly, Fontes Pereira et al. (2024) compared OD and conventional osteotomy in 90 patients and observed time-dependent increases in ISQ for both techniques [43]. However, factors such as implant length and bone arch were identified as influencing insertion torque in the OD group. The authors proposed that the compaction and “spring-back” effect of OD might enhance bone–implant contact and support osseointegration. After one year of follow-up, no statistically significant difference in stability was found, although OD did not adversely affect osseointegration [43]. These findings are generally consistent with the results of the present study, where the OD group showed slightly higher torque and ridge-width values, yet both techniques demonstrated a similar trend of stability increase over time. This pattern suggests that OD may confer mechanical advantages by preserving and compacting bone, though its contribution to long-term stability may be limited.

In a multicenter randomized controlled trial, Stacchi et al. (2022) [44] compared OD with piezoelectric implant site preparation (PISP) and found no significant differences in primary or secondary stability. Both methods exhibited an initial transient decrease in ISQ, followed by recovery after 90 days. These outcomes partially align with our observations. In the present study, significantly higher primary and secondary stability values were obtained in OD-prepared maxillary posterior regions than in conventional sites, whereas horizontal bone gain did not differ significantly. Taken together, both studies suggest that OD may improve early bone–implant contact and mechanical retention; however, this effect may not always translate into long-term stability or measurable volumetric gain. Stacchi et al.’s findings, limited to the posterior maxilla, may have been influenced by regional bone density and anatomic variation. In contrast, the present study examined both maxillary and mandibular regions, and OD appeared to offer more pronounced benefits in low-density bone. Thus, the advantages of OD may depend on bone density, implant design, and surgical protocol, highlighting the need for further standardized long-term clinical trials.

Fontes Pereira et al. (2023) conducted a systematic review encompassing 17 human and animal studies and concluded that OD generally provides higher primary stability and bone–implant contact (BIC) than conventional drilling [45]. Nevertheless, the authors emphasized that methodological heterogeneity and the limited availability of long-term randomized data constrain the generalizability of these findings. Consistently, in the present study, implants placed with OD burs exhibited higher primary and secondary stability in maxillary regions, whereas horizontal bone gain did not differ significantly between the techniques. These findings are generally consistent with the results obtained in the present study, where both techniques demonstrated a similar trend of stability increase over time. As also highlighted by Fontes Pereira et al., long-term, standardized clinical research is warranted to confirm these effects.

Bone regeneration and implant stability represent complex biological processes influenced by multiple factors beyond the drilling method. Previous studies have suggested that growth factors, collagen-based carriers, and resorbable biomaterials can modulate osteogenic activity and bone–implant interactions. Nocini et al. (2017) proposed that such adjuncts may enhance bone regeneration by supporting osteoblast activity during early healing [46]. Although concentrated growth factor (CGF) membranes were placed over all expanded alveolar ridges to ensure standardization across procedures, the stability differences observed in this study should not be attributed solely to the drilling technique but may also be influenced by surrounding biological factors and the host response.

### Limitations and Future Directions

This study has certain limitations. Its retrospective, single-center design and relatively short follow-up period (four months) limit causal inference and the generalizability of the findings. Furthermore, the use of a single implant system (Osstem) may restrict extrapolation to other implant geometries or drilling protocols. The retrospective design inherently carries risks of selection bias and record variability. Patient-reported outcomes (e.g., postoperative discomfort, complications, satisfaction) were not consistently documented and thus could not be analyzed. Additionally, potential confounders such as anatomical site, bone density, and operator variability were not standardized, preventing multivariate analysis. The absence of standardized CBCT HU data across all cases precluded direct quantitative comparison of baseline bone quality between groups. Nevertheless, the regional classification used (maxillary anterior/posterior, mandibular anterior/posterior) was deemed a clinically reasonable proxy, reflecting the typical D1–D4 bone density distribution described in the literature.

Future prospective, multicenter studies with longer follow-up periods are needed to validate these findings. Incorporating different implant systems and drilling protocols, as well as standardized evaluation of biological and patient-centered outcomes, may help clarify the long-term clinical implications of the OD technique.

## 5. Conclusions

Within the limitations of this retrospective study, the osseodensification (OD) technique appeared to exert a favorable influence on implant stability, particularly in low-density bone regions, while horizontal bone gain did not differ significantly from that achieved with conventional drilling. These findings suggest that OD may contribute to early mechanical stabilization without necessarily enhancing long-term volumetric outcomes. Clinically, OD may be considered a viable alternative for ridge-split and implant placement procedures; however, multicenter, prospective investigations encompassing various implant systems, bone densities, and extended follow-up periods are required to substantiate these preliminary observations.

## Figures and Tables

**Table 1:** Evaluation of Primary (T0) and Secondary Stability (T1) by Groups and Regions.

		OD Group		Control Group		*p*
		Mean ± Sd	Min–Max (Median)	Mean ± Sd	Min–Max (Median)	
Maxilla Anterior Region	T0 (Primary Stability)	81.98 ± 4	73–90 (82)	77.37 ± 5.5	65–86 (77)	^a^ 0.001 **
	T1 (Secondary Stability)	82.37 ± 4.91	73–92 (82)	77.27 ± 5.25	68–87 (77)	^a^ 0.001 **
Maxilla Posterior Region	T0 (Primary Stability)	81.54 ± 3.68	71–89 (82)	76 ± 5.63	62–86 (76)	^a^ 0.001 **
	T1 (Secondary Stability)	81.39 ± 4.55	72–89 (82)	74.61 ± 5.07	61–84 (75)	^a^ 0.001 **
Mandibula Anterior Region	T0 (Primary Stability)	81.6 ± 6.24	68–88 (84)	83.08 ± 4.81	74–88 (85)	^b^ 0.507
	T1 (Secondary Stability)	82.7 ± 4.99	75–92 (83)	85.17 ± 4.97	76–91 (86)	^b^ 0.290
Mandibula Posterior Region	T0 (Primary Stability)	83.93 ± 3.4	76–89 (85)	82.07 ± 5.25	71–90 (83)	^a^ 0.061
	T1 (Secondary Stability)	86 ± 4.25	71–94 (87)	81.41 ± 6.79	66–92 (83)	^a^ 0.001 **

^a^ Independent Samples Test ^b^ Mann–Whitney U Test ** *p* < 0.01.

**Table 2:** Evaluation of Follow-up Measurements Within Groups.

		T0 (Primary Stability)		T1 (Secondary Stability)		*p*
		Mean ± Sd	Min–Max (Median)	Mean ± Sd	Min–Max (Median)	
Maxilla Anterior Region	OD Group	81.98 ± 4.0	73–90 (82)	82.37 ± 4.91	73–92 (82)	^c^ 0.685
	Control Group	77.37 ± 5.5	65–86 (77)	77.27 ± 5.25	68–87 (77)	^c^ 0.934
Maxilla Posterior Region	OD Group	81.54 ± 3.68	71–89 (82)	81.39 ± 4.55	72–89 (82)	^c^ 0.845
	Control Group	76 ± 5.63	62–86 (76)	74.61 ± 5.07	61–84 (75)	^c^ 0.220
Mandibula Anterior Region	OD Group	81.6 ± 6.24	68–88 (84)	82.7 ± 4.99	75–92 (83)	^d^ 0.878
	Control Group	83.08 ± 4.81	74–88 (85)	85.17 ± 4.97	76–91 (86)	^d^ 0.271
Mandibula Posterior Region	OD Group	83.93 ± 3.4	76–89 (85)	86.0 ± 4.25	71–94 (87)	^c^ 0.021 *
	Control Group	82.07 ± 5.25	71–90 (83)	81.41 ± 6.79	66–92 (83)	^c^ 0.631

^c^ Paired-Samples Test ^d^ Wilcoxon Signed Rank Test * *p* < 0.05.

**Table 3:** Comparison of Ridge Widths (mm).

		Ridge Width (mm)				^a^ *p*
		Coronal		Apical		
		Mean ± Sd	Min–Max (Median)	Mean ± Sd	Min–Max (Median)	
Preop	OD Group	4.27 ± 1.03	3.1–5.8 (4.1)	7.12 ± 0.69	5.8–7.9 (7.2)	0.044 *
	Control Group	3.78 ± 1.12	2.7–5.8 (3.3)	6.52 ± 0.98	4.8–8.3 (6.7)	0.045 *
^b^ *p*		0.350		0.156		
Postop	OD Group	7.08 ± 0.58	6.3–7.9 (7.3)	7.86 ± 0.86	6.2–8.8 (8.1)	0.111
	Control Group	6.77 ± 0.97	5.9–8.7 (6.3)	7.73 ± 0.72	6.7–8.7 (7.8)	0.596
^b^ *p*		0.248		0.748		
Bone Gain	OD Group	2.81 ± 0.59	2–3.6 (3.1)	0.73 ± 0.2	0.4–1 (0.8)	0.001 **
	Control Group	2.99 ± 0.37	2.4–3.6 (3.1)	1.37 ± 0.83	0.4–2.8 (1.2)	0.001 **
^a^ *p*		0.720		0.156		

^a^ Mann–Whitney U Test ^b^ Independent Samples Test * *p* < 0.05; ** *p* < 0.01.

## Data Availability

The data supporting the findings of this study are not publicly available due to patient privacy restrictions.

## Abbreviations

The following abbreviations are used in this manuscript:

OD	Osseodensification
CGF	Concentrated Growth Factor
RFA	Resonance Frequency analysis
CBCT	Cone-Beam Computed Tomography
ISQ	Implant Stability Quotient
